# Primary Sclerosing Cholangitis: Diagnostic Criteria

**DOI:** 10.3390/tomography10010005

**Published:** 2024-01-07

**Authors:** Nora Cazzagon, Samantha Sarcognato, Elisa Catanzaro, Emanuela Bonaiuto, Matteo Peviani, Francesco Pezzato, Raffaella Motta

**Affiliations:** 1 Department of Surgery, Oncology and Gastroenterology, University of Padova, 35128 Padova, Italypezzatofrancesco94@gmail.com (F.P.); 2 Gastroenterology Unit, Azienda Ospedale—Università Padova, 35128 Padova, Italy; 3 Department of Pathology, Azienda ULSS2 Marca Trevigiana, 31100 Treviso, Italy; 4 Department of Cardiac, Thoracic, Vascular Sciences and Public Health—DCTV, University of Padova, 35128 Padova, Italy; raffaella.motta@unipd.it; 5 Radiology Unit, Azienda Ospedale—Università Padova, 35128 Padova, Italy

**Keywords:** PSC, rare liver disease, chronic cholestasis, magnetic resonance cholangiography, magnetic resonance imaging, liver biopsy, histological scoring system

## Abstract

Primary sclerosing cholangitis is a chronic cholestatic liver disease characterized by inflammation and fibrosis of intra- and/or extrahepatic bile ducts leading to the formation of multifocal strictures alternated to bile duct dilatations. The diagnosis of the most common subtype of the disease, the large duct PSC, is based on the presence of elevation of cholestatic indices, the association of typical cholangiographic findings assessed by magnetic resonance cholangiography and the exclusion of causes of secondary sclerosing cholangitis. Liver biopsy is not routinely applied for the diagnosis of large duct PSC but is mandatory in the case of suspicion of small duct PSC or overlap with autoimmune hepatitis.

## 1. Introduction

Primary sclerosing cholangitis is a chronic cholestatic liver disease characterized by inflammation and the fibrosis of intra and/or extrahepatic bile ducts leading to the formation of multifocal strictures alternated to bile duct dilatations [1]. The disease is rare but PSC represents a relevant cause of morbidity and mortality since it is often evolutive and it can lead to cirrhosis and its complications [2,3]. The only curative therapy in PSC patients is liver transplantation (LT), since no pharmacotherapy has proven effective to prevent disease progression. PSC is associated with inflammatory bowel disease (IBD) in up to 70–80% of patients, and, moreover, in patients with PSC, an increased risk of hepatobiliary and colorectal cancer (CRC), particularly in patients with concomitant IBD, has been reported [4]. Large duct PSC is the most frequent subtype of PSC but there are other subtypes including the PSC-autoimmune hepatitis (PSC-AIH) variant, small duct PSC and PSC with elevated IgG4. Autoimmune sclerosing cholangitis is another distinct nosological entity, characteristic of the pediatric population, which has been comprehensively reviewed in other sources [5]. The purpose of this review is to examine the diagnostic criteria employed in the diagnosis of primary sclerosing cholangitis (PSC) in adults.

The diagnosis of large duct PSC is mostly based on the presence of elevation of cholestatic indices and the association of typical cholangiographic findings, i.e., the presence of strictures and dilatations in the intra- and/or extrahepatic bile ducts after the exclusion of causes of secondary sclerosing cholangitis. These are shown in Table 1. In order to exclude other causes of sclerosing cholangitis, physicians should obtain a detailed medical history regarding the history of previous surgical interventions, HIV infection and/or risk factors for HIV infection, parasitic infections, recurrent cholangitis, personal or familial history of cholelithiasis in young age, intrahepatic cholestasis of pregnancy or cirrhosis of unknown origin and severe COVID-19 infection. In addition, current European and American guidelines recommend testing serum levels of IgG4 [6,7]. Finally, the presence of portal cavernoma, hilar lymphadenopathy and chronic pancreatitis should be excluded as well.

Magnetic resonance cholangiography (MRC) is the modality of choice to detect bile duct changes diagnostic of sclerosing cholangitis; since it is non-invasive, cost-effective [8] and has good sensitivity and specificity [9], it is indeed recommended by both European, UK and American guidelines as the first diagnostic modality in case PSC is suspected [6,7,10,11]. On the other hand, liver biopsy is recommended in the diagnostic pathway of PSC when the PSC-AIH variant or small duct PSC is suspected. 

## 2. Biochemical Features

The alteration of alkaline phosphatase in patients with IBD is the first index for suspecting PSC; however, cholestatic liver enzymes may spontaneously fluctuate in patients with PSC and could also be normal [12,13]. Serum alanine and aspartate aminotransferase are typically mildly elevated. On the other hand, an elevation of transaminases more than four to five times the upper limit of normal is typical during episodes of acute cholangitis or in patients with an overlap with AIH [14,15]. Serum bilirubin levels may increase in PSC due the development of benign or malignant severe strictures of extrahepatic bile ducts or in late stage PSC as an expression of hepatic dysfunction.

## 3. Serology

Positive titers of autoantibodies are common in PSC, including antinuclear antibodies (ANAs) in 8–77%, anti-smooth muscle antibodies (SMAs) in 0–83% and perinuclear anti-neutrophil nuclear antibody (p-ANCA) in 26–94% of patients [16,17,18]. Perinuclear-ANCAs are directed against myeloperoxidase, a cytoplasmic protein, while atypical p-ANCAs are directed against components of the nuclear envelope and have been identified in people with PSC but lack diagnostic specificity [19] and thus are not recommended by European and American guidelines for the diagnosis of PSC [6,7]. 

However, the clinical utility of detecting serum autoantibodies (ANA, SMA or LKM1) is limited to cases of the suspected PSC-AIH variant and contributes to the diagnosis together with the elevation of IgG serum levels, higher levels of transaminases and typical histological findings [6,7,20]. 

## 4. The Role of Magnetic Resonance Imaging in PSC Diagnosis

Magnetic resonance imaging (MRI) was introduced in the nineties in the diagnostic pathway of PSC, since it allows for a good visualization of the biliary tree and is non-invasive compared to endoscopic retrograde cholangiopancreatography (ERCP). The signal of bile ducts is increased by using T2-weighted (T2w) sequences resulting in increased contrast compared with the background and thus allowing a clear depiction of the bile ducts. Moreover, differently from ERCP, MRI allows anatomic imaging of extra ductal disease when magnetic resonance cholangiography (MRC) is combined with conventional T1- and T2w sequences. Other advantages of MRI are that (1) MRI is more cost-effective; (2) uses no ionizing radiation; (3) requires no anesthesia; (4) is less operator-dependent; and (5) better visualizes ducts proximal to a stricture or an obstruction. However, MRC shows a lower spatial resolution compared to ERCP, and indeed peripheral ductal abnormalities may not be visualized by MRC due their physiologic, non-distended state. A meta-analysis on the diagnostic performance of MRC in PSC reported a high sensitivity and specificity for the diagnosis of PSC, 86% and 94%, respectively, but without the risk of ERCP [9] and being cost-saving compared to ERCP [8].

For all these reasons, current guidelines recommend MRC as the primary diagnostic modality in the case of suspicion of PSC [6,7,10,11,21]. Moreover, MRI/MRC is indicated in patients with PSC in the following cases [6,7,10]: I. before therapeutic ERCP; II. in case of a suspicion of cholangiocarcinoma (CCA) before any invasive procedure; III. within 6 months of the diagnosis, including contrast media administration because of the higher risk of prevalent CCA when PSC is diagnosed [21]; IV. as a method for CCA screening [6,21].

## 5. Technical Aspects of MRI in PSC

MRC uses high-strength magnets (1.5–3 T) and, due to the high T2w signal intensity of bile, it provides a clear visualization of the biliary tree and the pancreatic duct. Fasting is a prerequisite and the oral administration of pineapple juice or diluted gadolinium contrast (1 mL in 200 mL of water) is recommended prior to MRC in order to suppress the signals of gastric and duodenal content. Three-dimensional (3D) MRC using 1 mm thickness slices is superior to two-dimensional (2D) MRCP because of a higher spatial resolution and an excellent signal/noise ratio and is favored for the diagnosis of PSC. 

Maximum intensity projection images (MIPs) and multiplanar reformatted images (MRP) obtained with the post-processing of images obtained with 1 mm thickness slices and suppressing noise enable the spatial visualization of the biliary tree and the evaluation of small abnormalities in bile ducts, too [22]. Meanwhile, 2D breath-hold thick slab sequences have a shorter acquisition time, thus limiting the occurrence of motion artefacts in patients with low compliance for breath hold and immediate interpretation without needing post-processing [22]. 

T1-weighted (T1w) fat-suppressed sequences are also acquired to complete the MRI of the biliary tree and are able to detect the presence of intrahepatic calculi, as well as the presence of liver dysmorphy, signs of portal hypertension and splenomegaly. Moreover, the use of gadolinium-based contrast agent (GBCA) sequences is useful for providing information on the biliary wall and liver parenchyma. There are two types of GBCAs that are used in the setting of biliary diseases, including hepatospecific agents (e.g., Gadobenate dimeglumine, Gd-BOPTA, MultiHance, Bracco, Gd-EOB-DTPA, Primovist or Eovist, Bayer) and extracellular agents (e.g., Gadoterate meglumine, Gd-DOTA, Dotarem, Guerbet, Gadopentetate Dimeglumine, Gd-DTPA, Magnevist, Schering AG) [23]. 

In contrast to extracellular agents, which are primarily eliminated through renal filtration, approximately half of the GD-EOB-DTPA is rapidly taken up by hepatocytes and excreted into the bile. This results in a robust delayed imaging of the hepatic and biliary tree. In individuals with normal liver function and without parenchymal disease, the enhancement of the biliary tree with these agents is typically sufficient to accurately visualize small intrahepatic ducts up to third-order branching. Conversely, Gd-BOPTA experiences only 3–5% biliary excretion. 

While T2w-MRCP offers higher spatial resolution in peripheral ducts compared to both T1w-MRCP [24,25] and ERCP [26], T1w-MRCP provides more precise visualization of central structures than T2w-MRCP [25]. It can also contribute diagnostic information regarding parenchymal function.

The use of hepatospecific GBCA and dynamic T1w sequences, including the hepatospecific phase, allows for the visualization of local or diffuse impairments of liver parenchyma and alterations in the contrast excretion in central bile ducts [24]. In patients with PSC, the excretion of hepatospecific GBCA is often delayed compared to healthy controls and correlates with hepatic function, as estimated by bilirubin levels [26].

Furthermore, T1w fat-suppressed sequences before and after GBCA injection may reveal biliary wall thickening and mural enhancement of the biliary ducts after injection, as well as wedge-shaped alterations in the liver parenchyma.

These alterations, caused by confluent fibrosis, typically appear hypointense in T1w sequences in the pre-contrast phase compared to the surrounding parenchyma. They increase in signal during arterial and portal phases. In the delayed phase, their signal intensity continues to increase with extracellular agents, while with hepatospecific agents, they become hypointense compared to the surrounding parenchyma and further decrease in intensity in the hepatospecific phase. This pattern is attributed to the lower presence of hepatocytes within the confluent fibrotic area, accumulating a higher quantity of contrast in the hepatospecific phase compared to the focal fibrotic area [27]. These wedge-shaped areas have also been visualized using T2w sequences before and after GBCA injection with the quantification of relative liver enhancement [28].

Although several studies have aimed to determine whether dynamic MRI with hepatospecific GBCA can discriminate between different stages of fibrosis, no definitive results have been provided [29,30].

The use of contrast agents is not obligatory for PSC diagnosis but is recommended when suspecting CCA or within the first six months of PSC diagnosis to enhance diagnostic performance in prevalent CCA cases. The International PSC Study Group’s position statement outlines a minimum and complete standard protocol for the diagnostic workup of suspected PSC patients [21].

The minimum standard protocol includes T2-weighted MRCP (preferably 3D over 2D-MRCP) and T1- and T2-weighted axial sequences for liver parenchyma visualization [21]. The complete protocol, incorporating sequences after GBCA injection, comprises T1 contrast dynamic sequences with arterial, portal venous, and parenchymal phases, T1-weighted MRCP and T1-weighted hepatobiliary phases (the latter two when hepatospecific contrast agents are used) [21]. Currently, there is no evidence favoring the use of extracellular or hepatospecific GBCA in PSC patients.

Finally, additional MR techniques could augment information from classic MRI sequences and may be included in the MRI protocol. Magnetic resonance elastography allows for the assessment of liver fibrosis throughout the entire parenchyma [21,31,32]. Moreover, diffusion-weighted imaging (DWI) could also be included in the complete protocol of MRI in patients with PSC [21]. A recent study suggests that DWI correlates with fibrosis assessed by transient elastography but may not distinguish moderate/severe fibrosis (F2–F3) from cirrhosis (F4) [29].

## 6. Cholangiographic and Liver Parenchymal Changes in PSC

The initial documentation of cholangiographic observations in PSC through ERCP was published in 1983. This study encompassed 86 patients diagnosed with PSC, 16 with primary biliary cirrhosis (PBC) and 82 with primary bile duct carcinoma [33].

**Figure 1:**
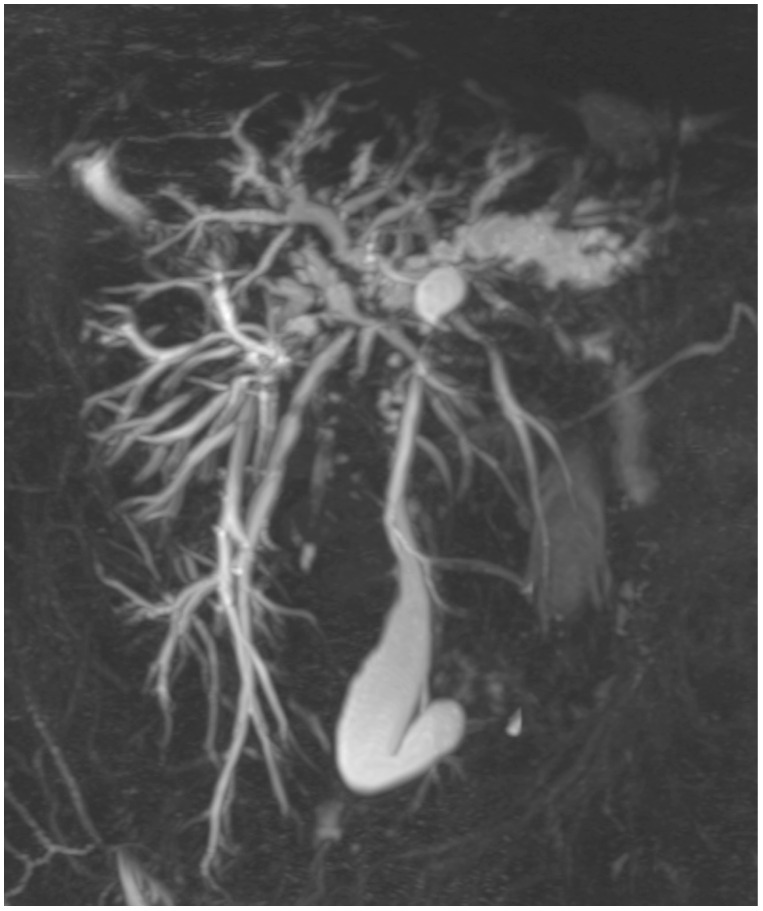
Primary sclerosing cholangitis. The 3D-MRC shows the presence of multiple strictures and dilatation of intra- and extrahepatic bile ducts. The common bile duct is severely stenotic in all its length.

The predominant discovery was the prevalence of multifocal strictures (depicted in Figure 1), affecting both intra- and extrahepatic bile ducts, which was more frequently observed in PSC compared to the other groups. The characteristic “beaded” appearance of the bile ducts was described, featuring diffusely distributed, short, annular strictures interspersed with normal or slightly dilated segments [33].

Additionally, the authors noted the occurrence of band-like strictures, diverticulum-like out-pouching and diverticula without band strictures in 20%, 10% and 16% of patients with PSC, respectively [33]. Furthermore, abnormalities in the pancreatic duct were recorded in 3 out of 40 patients.

Subsequent investigations have amalgamated these cholangiographic observations to categorize distinct radiological subtypes of the disease [34,35]. Majoie et al. introduced one of the initial descriptive classifications for bile duct changes [34], which was later refined by Ponsioen et al. through the addition of the category 0 to differentiate between intra- and extrahepatic disease. This classification was then employed to establish the Amsterdam cholangiographic score (refer to Table 2) [36].

In that very year, Craig et al. introduced a quantitative categorization of anomalies in the bile duct (see Table 3). The merit of this classification lies in the incorporation of objective criteria to delineate various cholangiographic observations and the precise localization of the lesions [37].

**Figure 2:**
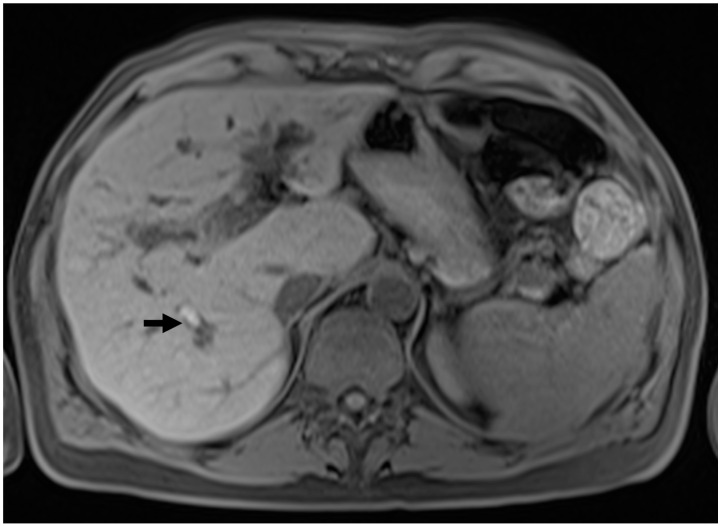
Intraductal stones in PSC. T1w pre-contrast sequence showing the presence of a hyperintense calculus in a dilated intrahepatic bile duct in the posterior segment.

Additional cholangiographic observations in PSC include the identification of primary pigmented intraductal stones (depicted in Figure 2), observed in as many as 30% of PSC patients [38]. Furthermore, certain authors have proposed that a retracted papilla, visualized through either ERCP or MRCP, serves as a specific indication of PSC. However, these findings have yet to be validated in external cohorts [39,40].

**Figure 3:**
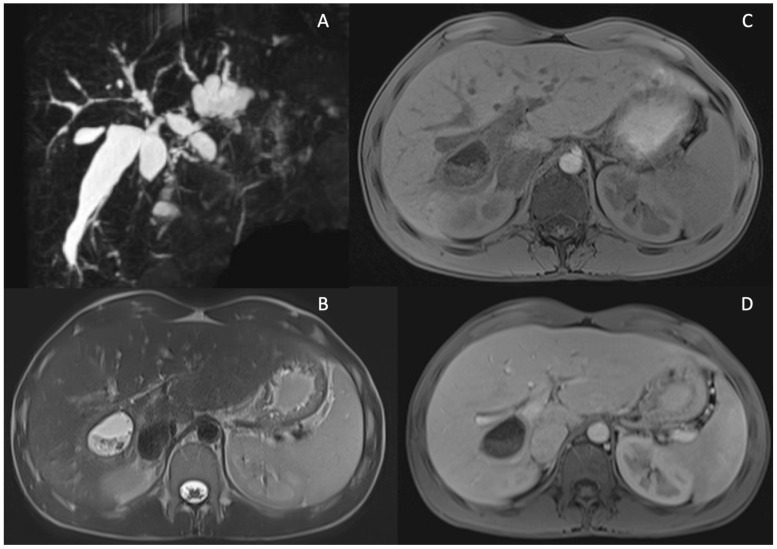
Cystic dilatations (CD) of intrahepatic bile ducts in PSC patients. MRCP sequence showing diffuse cystic dilatations of intrahepatic bile duct (**A**). MRI showing a large CD of intrahepatic bile duct dilatation localized between S5 and S6 filled with stones in T2 w images (**B**), T1w fat sat pre-contrast (**C**) and after Gd-EOB-DTPA (portal phase) showing wall thickening and enhancement of the CD (**D**).

Lastly, the occurrence of biliary cystic dilatation (illustrated in Figure 3) within the intrahepatic bile ducts was recorded in 21 patients diagnosed with PSC [41,42,43,44,45,46,47] as well as in five livers that underwent explantation [48].

**Figure 4:**
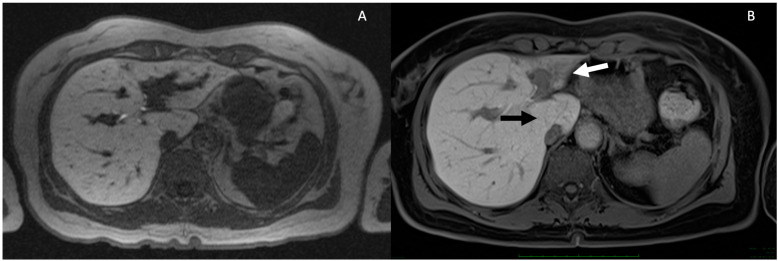
Progressive parenchymal atrophy in PSC in the hepatobiliary phase. Severe intrahepatic BD stricture and dilatation in S2–S3 lobe is observed (**A**). Eight years later, the patient developed a severe atrophy of S2–S3 (white arrow) and hypertrophy of S1 (black arrow) (**B**).

The initial documentation of magnetic resonance (MR) findings in a small group of PSC patients dates back to the late nineties. Applying the Majoie classification of bile duct anomalies to MRI, the authors noted a 5% overestimation of intrahepatic disease and a 10% discrepancy, both over- and underestimation, of extrahepatic disease compared to 57 cases assessed through ERCP [49]. Subsequent studies have explored the utility of MRCP in PSC, consistently confirming its high diagnostic accuracy [24,26,50,51,52]. As previously mentioned, the advantage of MRI over ERCP lies in its ability to delineate morphological alterations in the liver parenchyma. The identification of peripheral wedge-shaped areas of parenchymal atrophy, also known as confluent focal fibrosis or focal atrophy (depicted in Figure 4), has been widely reported. Importantly, these areas are not always associated with the cirrhotic stage [53]. In fact, focal atrophy likely arises as a consequence of the progression of focal fibrosis surrounding bile ducts, leading to duct obliteration and the subsequent impairment of cholestasis and inflammation and the development of focal fibrosis in the liver parenchyma [22].

**Figure 5:**
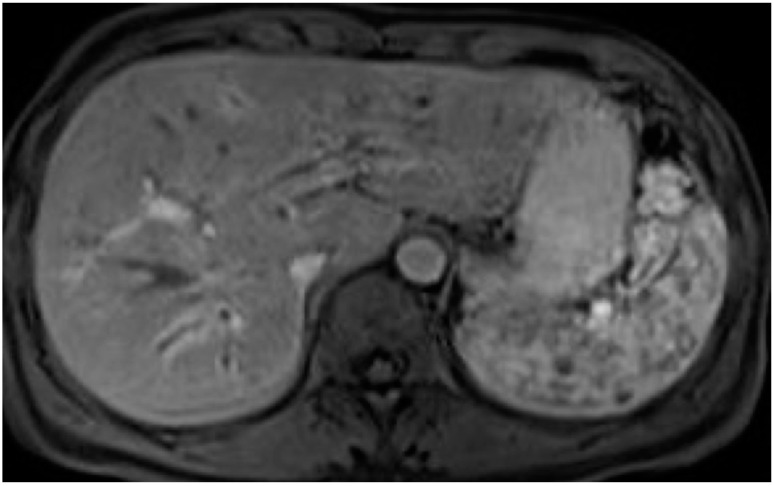
Biliary wall thickening and mural enhancement of the intrahepatic biliary ducts after contrast. T1-weighted sequences after Gd-EOB-DTPA injection.

The existence of thickening in the biliary walls and mural contrast enhancement of the biliary ducts (as shown in Figure 5) has been noted in reports, and there is a potential link to inflammatory processes [22].

**Figure 6:**
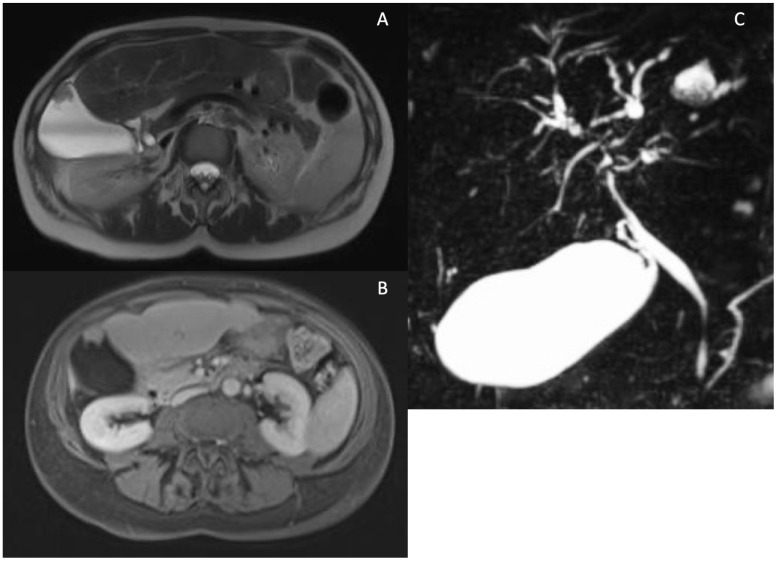
Possible gallbladder findings in PSC. Gallbladder mass observed in T2 weighted (**A**) and T1 weighted after contrast images (**B**). MRCP showing an enlarged gallbladder in PSC (**C**).

Abnormalities in the gallbladder have been observed in 41% of patients with PSC [54,55]. These abnormalities include the presence of gallstones in one-fourth of patients, wall thickening independent of portal hypertension in 15% of patients and mass lesions in 4–6% of patients (refer to Figure 6). In PSC patients, mass lesions in the form of polyps are malignant in 56–57% of cases [54,56], underscoring the rationale for annual ultrasound surveillance in these patients [6,7].

Furthermore, when gallbladder polyps exceed 8 mm, cholecystectomy is recommended according to European guidelines [6], while consideration should be given according to American guidelines, particularly at experienced centers for patients with advanced disease [7]. Lastly, an increase in pre- and post-prandial gallbladder volume (as shown in Figure 6) compared to healthy controls has been observed in PSC patients [57,58]. The reason for this volume increase has not been fully explained. Nevertheless, a recent French study indicated that an enlarged gallbladder in PSC patients is associated with reduced cholestasis and lower levels of hydrophobic serum bile acids. In contrast, cholecystectomized patients exhibit more severe cholangiographic features, suggesting the potentially protective role of an enlarged gallbladder in PSC [59].

A significant advantage of MRI in primary sclerosing cholangitis (PSC) is its capability to systematically monitor disease progression, which occurs in approximately 60% of patients over a median follow-up period of 4 years [60].

In their investigation, Ruiz et al. employed a standardized model for interpreting MRI findings (refer to Table 4), quantifying rather than describing the disease’s severity. This approach enabled the authors to identify specific radiological features that are independently associated with disease progression, including dysmorphy, severity of intrahepatic bile duct dilatation, signs of portal hypertension and parenchymal enhancement heterogeneity [60].

These features were incorporated into two scores, termed ANALI scores, with or without gadolinium injection, depending on the presence of sequences after the administration of the contrast agent [60]. Subsequently, these straightforward scores were proven to be valuable for prognostic purposes in PSC patients when used either in isolation [61] or in conjunction with liver stiffness evaluated through vibration-controlled transient elastography [62].

Despite the efforts to standardize the MRI imaging protocol for suspected PSC, the interpretation of PSC changes remains challenging even for expert radiologists, as reported by Zenouzi and colleagues [63]. For these reasons, Ringe and colleagues developed a deep learning algorithm for the automated detection of PSC-compatible cholangiographic changes in 3D-MRC images in a dataset of 428 patients, half of them with PSC and the other half non-PSC [64]. They reported a great accuracy in detecting and excluding PSC as confirmed by reported values of sensitivity, specificity, positive predictive value and negative predictive value all higher than 90% [64]. More recently, Ragab and Westhaeusser proposed a deep learning model for the automated diagnosis of PSC using 2D-MRC imaging in a dataset of 342 PSC patients and 264 controls and reported an overall accuracy of 80.5% [65].

Recently, a post-processing tool (MRCP+™, Perspectum diagnostic) able to provide a semi-automatic quantification of the extent and severity of bile duct stricture was suggested as a possible prognostic tool in PSC [66,67], but further data are needed to clarify the association between MRCP+ metrics and prognosis.

**Figure 7:**
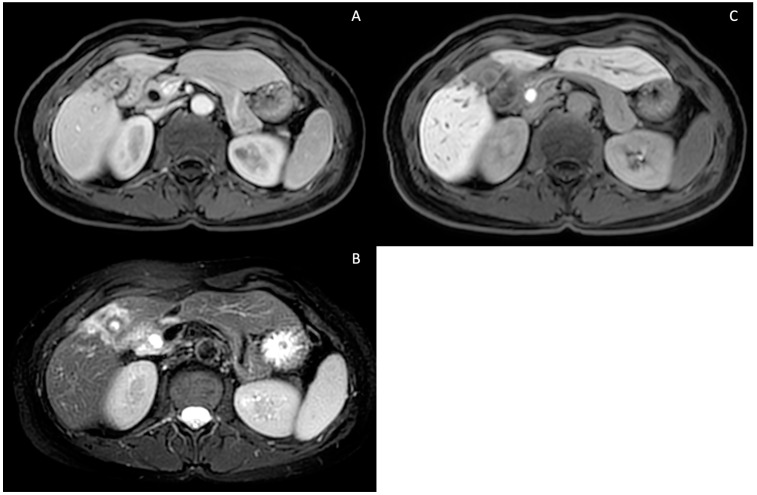
**Intrahepatic pericholecystic cholangiocarcinoma**. T1-weighted contrast-enhanced portal phase (**A**), T2-weighted sequence showing hyperintensity due to increased cellularity (**B**) and T1-weighted contrast-enhanced (Gd-EOB-DTPA) hepatospecific phase showing hypointensity due to a lack of hepatocellular contrast uptake (**C**).

Cholangiocarcinoma (see Figure 7) represents an unpredictable complication of PSC and does not necessarily correlate with advanced disease stages. No cholangiographic features are pathognomonic for CCA, and while the majority of CCAs occur in the common hepatic duct or at the bifurcation (Klatskin’s tumor), some originate in the intrahepatic bile ducts. However, there is limited information available regarding the localization of CCA in PSC patients. According to MacCarty et al., cholangiographic indications suggestive of CCA include irregular high-grade ductal narrowing with irregular edges, rapid stricture progression, significant ductal dilatation proximal to strictures and the presence of polypoid lesions, particularly those larger than 1 cm in diameter [68]. In comparison to ERCP, both MRI and computed tomography offer the advantage of assessing the extra ductal extent, enabling better tumor staging [38]. Additionally, as many CCAs have a fibrous core, delayed enhancement and washout after gadolinium-based contrast agent (GBCA) injection are nearly 100% specific for CCA [21]. Cross-sectional imaging is crucial for CCA diagnosis and evaluating its resectability, with MRI proving superior to CT in cholangiocarcinoma detection [69]. Furthermore, the addition of GBCA during MRI enhances the sensitivity of the technique for CCA detection [70].

## 7. Liver Biopsy

Liver biopsy is no longer routinely performed to establish a diagnosis of PSC [11,71,72], but it is mandatory in case of suspicion of small duct PSC or whenever a PSC-AIH variant or the presence of other comorbidities is clinically suspected [73]. Moreover, liver biopsy is also adopted in the clinical trial setting as a valuable method for risk stratification and as a surrogate endpoint for clinical trials [74]. Indeed, strong evidence confirms that histological staging is strongly and independently associated with prognosis in PSC patients [75,76].

**Figure 8:**
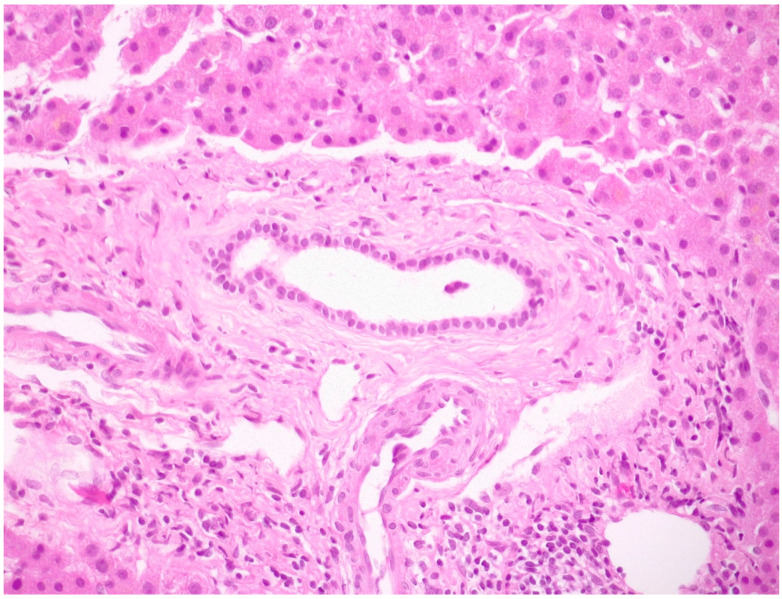
A medium-size intrahepatic bile duct with concentric periductal fibrosis (i.e., onion skin fibrosis) in a PSC biopsy (hematoxylin–eosin; original magnification 20×).

A typical histological feature of PSC is the presence of an inflammatory infiltrate in a large intra- and extra-hepatic bile duct wall associated with an obliterative concentric periductal loose fibrosis called “onion skin fibrosis” (Figure 8).

These lesions could lead to biliary strictures and eventually occlusions called bile duct scars [73]. In early stages of the disease, histological alterations are limited to portal tracts with the presence of a mild mixed inflammatory cell infiltrate consisting of lymphocytes, plasma cells and neutrophils, usually more intense around bile ducts. Lymphoid follicles or aggregates may also be seen, while the presence of lymphocytic interface hepatitis and/or lobular infiltrates may suggest coexistent AIH [20,48,73,77,78]. In the appropriate clinical context, fibro-obliterative duct lesions, although present only in a minority of cases [48] might be diagnostic of PSC, and the histological appearance of PSC may vary from normal liver tissue to the presence of only indirect signs of large bile duct obstruction [73].

During the disease course, there is a progressive loss of small- and medium-sized bile ducts (ductopenia) associated with the development of secondary changes related to chronic cholestasis, including ductular reaction, hepatocyte metaplasia and the deposition of copper and copper-binding proteins, such as orcein, in periportal hepatocytes [73]. The persistence of an inflammatory insult leads to portal and periportal fibrosis, which may evolve to bridging fibrosis and ultimately to the development of biliary cirrhosis, characterized by edema at the periphery of the fibrous septa, which gives rise to the typical halo effect [73]. Other frequent findings reported in a series of 15 explanted livers of PSC patients are the presence of inflammation, ulceration and ectasia of large intrahepatic ducts [48].

The histological staging of PSC was classically assessed using the system proposed by Ludwig and colleagues [79] that recognizes the presence of four histological stages. The portal stage (I) was characterized by the presence of portal inflammation, connective tissue expansion and cholangitis, limited to the portal tracts. The periportal stage (II) was defined by the presence of an inflammatory infiltrate (interface hepatitis) and fibrosis (periportal fibrosis) beyond the limiting hepatocyte plate. The septal stage (III) was characterized by bridging fibrosis, while the cirrhotic stage (IV) consisted of parenchymal nodules formed by bands of fibrosis [79]. More recently, the Nakanuma system for PBC staging [80] was applied to PSC, where it showed a strong association with prognosis [75,76]. According to the Nakanuma system, grading is determined by the extent of chronic cholangitis activity (CA) and hepatitis activity (HA), while for stage assessment, a two-criteria (extent of fibrosis and bile duct loss) or three-criteria (with the addiction of orcein-positive granule deposition evaluation) method could be applied [81] (Table 5).

## 8. Conclusions

In conclusion, the diagnosis of large duct PSC, the most common subtype of the disease, is based on the presence of elevation of cholestatic indices and the association with typical cholangiographic findings, i.e., the presence of strictures and dilatations in the intra- and/or extrahepatic bile ducts after the exclusion of causes of secondary sclerosing cholangitis. Magnetic resonance imaging is the first imaging choice at diagnosis and is very useful to monitor biliary and parenchymal changes and the occurrence of complications during the course of the disease. Liver biopsy is not routinely performed for the diagnosis of large duct PSC but is mandatory in the case of suspicion of small duct PSC or overlap with AIH. Differently, in the clinical trial setting, liver biopsy is widely used for risk stratification at inclusion and as a surrogate endpoint, since the histological stage has a strong and independent prognostic value.

## Figures and Tables

**Table 1:** Causes of secondary sclerosing cholangitis.

Causes of Secondary Sclerosing Cholangitis
Cholangiocarcinoma
IgG4-related sclerosing cholangitis
HIV infection
Choledocholithiasis
Sarcoidosis
Traumatic or ischemic biliary injury
Papillary stenosis
Ampullary or pancreatic cancer
Chronic pancreatitis
Hilar lymphadenopathy
Congenital
Chronic biliary infestation
Recurrent pyogenic cholangitis
Portal biliopathy
ABCB4 deficiency
Post-COVID-19 sclerosing cholangitis

**Table 2:** Classification of cholangiographic findings in PSC according to Majoie and modified by Ponsioen et al.

Type of Duct Involvement/Classification	Cholangiographic Abnormalities
Intrahepatic	
0	No visible abnormalities
I	Multiple caliber changes; minimal dilatation
II	Multiple strictures, saccular dilatation, decreased arborization
III	Only central branches filled despite adequate filling pressure; severe pruning
**Extrahepatic**	
0	No visible abnormalities
I	Slight irregularities of duct contour; no stricture
II	Segmental stricture
III	Stricture of almost entire length of duct
IV	Extremely irregular margin; diverticulum-like outpouchings

**Table 3:** Classification of cholangiographic findings in PSC according to Craig et al.

Characteristic	Classification
Bile duct strictures	
**Grade**	1: 0–25% narrowing of duct 2: >25–50% narrowing of duct 3: >50–75% narrowing of duct 4: >75–100% narrowing of duct
**Length**	Band: 1–2 mm of involvement Segmental: 3–10 mm of involvement Confluent: >10 mm of involvement
**Extent**	Localized: ≤25% of duct involvement Diffuse: >25% of duct involvement
Bile duct dilatation	
**Common bile duct**	None: <15 mm Mild: 15–19 mm Marked: ≥20 mm
**Left main hepatic duct**	None: <7 mm Mild: 7–9 mm Marked: ≥10 mm
**Right main hepatic duct**	None: <6 mm Mild: 6–7 mm Marked: ≥8 mm
**Secondary intrahepatic ducts**	None: <4 mm Mild: 4 mm Marked: ≥5 mm

**Table 4:** Standard model of interpretation by Ruiz et al. [60].

Feature	Points
CBD stricture	0 = no stricture; 1 = stricture ≤ 75%; 2 = stricture > 75%
CBD stricture length	0 = absent; 1 = band (stricture < 2 mm); 2 = segmental (stricture 2–10 mm); 3 = confluent (stricture > 10 mm)
CBD dilatation	0 = none (≤10 mm); 1 = mild (11–14 mm); 2 = marked (≥15 mm)
CBD enhancement	0 = absent; 1 = thickness < 2 mm; 2 = thickness 2–6 mm; 3 = thickness > 6 mm
RHD stricture	0 = no stricture; 1 = stricture ≤ 75%; 2 = stricture > 75%
RHD stricture length	0 = absent; 1 = band (stricture < 2 mm); 2 = segmental (stricture 2–10 mm); 3 = confluent (stricture >10 mm)
RHD dilatation	0 = none (≤6 mm); 1 = mild (7–8 mm); 2 = marked (≥9 mm)
RHD enhancement	0 = absent; 1 = thickness < 2 mm; 2 = thickness 2–6 mm; 3 = thickness > 6 mm
LHD stricture	0 = no stricture; 1 = stricture ≤ 75%; 2 = stricture > 75%
LHD stricture length	0 = absent; 1 = band (stricture < 2 mm); 2 = segmental (stricture 2–10 mm); 3 = confluent (stricture > 10 mm)
LHD dilatation	0 = none (≤6 mm); 1 = mild (7–8 mm); 2 = marked (≥9 mm)
LHD enhancement	0 = absent; 1 = thickness < 2 mm; 2 = thickness 2–6 mm; 3 = thickness > 6 mm
IHBD stricture	0 = no stricture; 1 = stricture ≤ 75%; 2 = stricture > 75%
IHBD liver involvement	0 = absent; 1 = localized (≤25% IHBD involved); 2 = diffuse (>25% IHBD involved)
IHBD dilatation	0 = none (≤3 mm); 1 = mild (4 mm); 2 = marked (≥5 mm)
IHBD enhancement	0 = absent; 1 = thickness < 2 mm; 2 = thickness 2–6 mm; 3 = thickness > 6 mm
Parenchymal enhancement heterogeneity	0 = absent; 1 = present
Intraductal stones	0 = absent; 1 = present
Dysmorphy	0 = absent; 1 = present
Portal hypertension	0 = absent; 1 = present
ANALI score without gadolinium	1 × IHBD dilatation + 2 × Dysmorphy + 1 × Portal hypertension
ANALI with gadolinium	1 × Dysmorphy + 1 × Parenchymal enhancement heterogeneity

Abbreviations: CBD, common bile duct; RHD, right hepatic duct; LHD, left hepatic duct; IHBD, intrahepatic bile ducts. Definitions: portal hypertension was defined by the presence of portosystemic shunts with or without splenomegaly.

**Table 5:** Grading and staging system according Nakanuma.

Grading System	
Chronic cholangitis activity (CA)	
CA 0 (no activity)	No cholangitis, but mild biliary epithelial damage may be present
CA 1 (mild activity)	1 bile duct (BD) with evident chronic cholangitis
CA 2 (moderate activity)	≥2 BDs with evident chronic cholangitis
CA 3 (marked activity)	≥1 bile duct with chronic non-suppurative destructive cholangitis (florid duct lesion)
Hepatitic activity (HA)	
HA 0 (no activity)	No interface hepatitis (IH) and no or minimal lobular hepatitis (LH)
HA 1 (mild activity)	IH affecting ≥ 10 continuous hepatocytes in 1 portal tract (PT) or fibrous septa, and mild-to-moderate LH
HA 2 (moderate activity)	IH affecting ≥ 10 continuous hepatocytes in ≥2 PTs or fibrous septa, and mild-to-moderate LH
HA 3 (marked activity)	IH affecting ≥ 20 continuous hepatocytes in ≥1/2 PTs, and moderate LH or bridging or zonal necrosis
Staging System	
Fibrosis	
0	No portal fibrosis or fibrosis limited to PTs
1	Portal fibrosis with periportal fibrosis and/or incomplete fibrous septa
2	Bridging fibrosis with variable lobular disarray
3	Liver cirrhosis with regenerative nodules and extensive fibrosis
Bile duct loss	
0	No bile duct loss (BDL)
1	BDL in less than one third of PTs
2	BDL in one third to two thirds of PTs
3	BDL in more than 2/3 of PTs
Deposition of orcein positive granules	
0	No deposition of orcein-positive granules (DOG)
1	DOG in few periportal hepatocytes in less than one-third of PTs
2	DOG in several periportal hepatocytes in one-third to two-thirds of PTs
3	DOG in many hepatocytes in more than two-thirds of PTs
Stage	2 criteria (fibrosis and bile duct loss)
Stage 1 (no progression)	0
Stage 2 (mild progression)	1–2
Stage 3 (moderate progression)	3–4
Stage 4 (advanced progression)	5–6
Stage	3 criteria (fibrosis, bile duct loss and deposition of orcein-positive granules)
Stage 1 (no progression)	0
Stage 2 (mild progression)	1–3
Stage 3 (moderate progression)	4–6
Stage 4 (advanced progression)	7–9
